# Optimization of Multi-gravity Separator (MGS) Parameters for Specularite Ore Beneficiation: Evaluating Box–Behnken and Central Composite Designs

**DOI:** 10.1021/acsomega.5c10890

**Published:** 2026-03-12

**Authors:** Çağrı ÇERİK, Sezai ŞEN

**Affiliations:** 37508 Dokuz Eylul University, Department of Mining Engineering, Izmir 35390, Turkey

## Abstract

This study investigates the optimization of multigravity separator (MGS) parameters for the beneficiation of specularite ore, using both Box–Behnken design (BBD) and central composite design (CCD) as response surface methodologies. The effects of the drum speed, wash water amount, and drum slope on the grade and recovery of specularite were evaluated. Results indicated that increased wash water and drum slope generally enhanced the Fe grade, while higher drum speeds had adverse effects. Conversely, recovery was maximized with lower wash water amounts, lower drum slopes, and higher drum speeds. The comparison between BBD and CCD designs revealed that both methods provided similar optimal grade and recovery values, though with different parameter settings. CCD demonstrated superior predictive performance with higher *F*-values and closer agreement between adjusted and predicted *R* ^2^. Multiresponse desirability optimization identified optimum conditions of approximately a 143 rpm drum speed, a 6.2 L/min wash water, and a 4.5° drum slope for CCD, achieving a composite desirability of 0.891. Confirmation tests closely matched predicted values with <1% deviation, validating model accuracy. The optimized conditions yielded a specularite concentrate assaying 63.97% Fe with 72.05% recovery. The findings demonstrate that MGS, when appropriately optimized, can effectively upgrade specularite ores to meet the quality requirements of micaceous iron oxide (MIO) pigment production. This work provides the first comparative assessment of BBD and CCD for MGS-based specularite beneficiation and establishes a validated optimization framework that integrates mineralogical, statistical, and operational insights.

## Graphical abstract

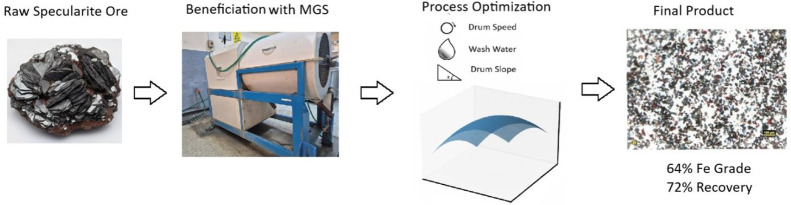

## 1 Introduction

Specularite (also known as micaceous iron oxide or specular hematite) is a variety of hematite characterized by its silvery color, dense and smooth surface, and metallic luster, forming as sheet-like aggregates with a theoretical iron content of 70% wt.^1,2^ The chemical composition of specular hematite is identical to that of hematite (Fe_2_O_3_), but its crystals manifest as gray platelets. This crystal structure represents the most stable form of hematite.^3^ Specularite has a characteristic planar structure, making it useful in various industries, such as welding electrodes, paint and coating, and brake pads.^4^ Micaceous iron has garnered significant attention for its durability, excellent chemical stability, and cost efficiency.^5–8^ Specularite is an excellent pigment in paints, offering physical protection for steel constructions.^9–11^ The lamellar pigment particles align as an overlapping array of parallel plates on the surface, providing a barrier protection against corrosive substances and water.^12–16^

Raw specularite ore might have a relatively low iron grade or contain various compounds and gangue minerals. In order to use specularite in industries such as welding and coating with the required standard characteristics, it must be subjected to enrichment processes. Similar to other types of iron ores, the most commonly proposed and applicable processes for specularite beneficiation include gravity concentration methods, magnetic separation, and flotation.

Flotation is considered to be an effective and widely used method of iron ore beneficiation.^17–19^ Researchers have studied the flotation of iron minerals with anionic and cationic collectors.^20–24^ Anionic flotation is a very attractive method for enriching iron ore associated with iron-bearing silicate minerals, but its enrichment efficiency is poor due to the low selectivity of flotation reagents.^24,25^ Under the same physicochemical conditions, quartz always exhibits greater floating capabilities than magnetite and hematite.^26^ Therefore, reverse cationic flotation is commonly used for beneficiation of iron-bearing oxides.^27–29^

High-gradient magnetic separators (HGMS) are effective devices for recovering fine, weakly magnetic particles from ores.^30^ Previous studies have shown that magnetic separators have been used for the enrichment of specularite ore successfully.^31–35^ Furthermore, researchers used magnetic separators after roasting and flocculation processes to increase magnetic recovery efficiency, yielding promising results.^36,37^ Although enhanced gravity separators are used for beneficiation of different types of iron ores,^38–44^ there are only a limited number of studies on the gravity separation of specularite ore in the literature.^2,4,33,34^

Enhanced gravity separators including the Falcon concentrator, multigravity separator (MGS), Kelsey jig, Altar jig, and Knelson concentrator operate by applying centrifugal force to improve the efficiency of conventional gravity separation. Among these devices, the multigravity separator (MGS) has gained widespread acceptance, particularly for fine particle processing in mineral and coal studies. As a specialized flowing-film concentrator, the MGS offers high separation performance for ultrafine particles, making it suitable for ores such as specularite.

Because MGS performance depends on multiple interacting variables, previous modeling efforts have primarily focused on identifying optimal operating conditions using regression-based approaches.^45–51^ To optimize these complex systems, researchers have applied various techniques, including response surface methodology (RSM), artificial neural networks (ANN), and extreme learning machines (ELM), with RSM being the most widely used. RSM encompasses statistical design tools such as Box–Behnken design (BBD) and central composite design (CCD), both of which are frequently employed for process optimization. Analysis of variance (ANOVA) is typically applied to evaluate the significance of parameters and develop predictive mathematical models within these frameworks.

To the best of our knowledge, there are no reports on the application of the Box–Behnken design for optimizing the multigravity separator (MGS) parameters for specularite ore. Furthermore, there are no reports comparing central composite design (CCD) and Box–Behnken design as multivariate statistical methods in mineral processing. Therefore, the purpose of this study is to optimize the operational parameters of the multigravity separator (MGS) and compare the performance of the Box–Behnken design (BBD) with the central composite design (CCD) to select the best method for optimizing the process parameters.

## 2 Materials and Methods

### 2.1 Materials

A specularite ore sample from Manisa, Turkey, was utilized in the study. The sample was crushed to below 1 mm (*d*_80_) by using a laboratory jaw crusher. Representative samples were taken from the crushed material and prepared for grinding. The grinding process was carried out using a laboratory-scale rod mill, and the material was ground to a particle size of 106 μm. The grinding target (<100 μm) was selected primarily to approach liberation for fine specularite–gangue intergrowths and to provide a feed size compatible with enhanced gravity separation using the multigravity separator (MGS), which is widely applied to fine/ultrafine particles. In addition, micaceous iron oxide (MIO) pigments are commonly supplied as fine powders with sieve-based specifications in pigment standards; therefore, <100 μm also represents a realistic industrial fineness window for the intended product form.^4,13,52^ Wet sieve analysis was conducted to determine the particle size distribution of the ground material, employing a vibratory sieve shaker and micro-precision sieves. The results of the size-wise chemical analysis are presented in Table 1.

**Table 1:** Size-Wise Chemical Analysis Results of Specularite

Particle size (μm)	wt (%)	Fe (%)
106–75	25.27	48.62
75–53	14.46	50.13
53–38	15.58	49.71
38–25	12.36	51.06
–25	32.33	51.59
total	100	50.27

The size-wise Fe assays show only a modest variation among fractions. Limited grade stratification by size can occur when specularite and gangue are finely intergrown, and breakage is not strongly selective within the investigated size range. Therefore, it is evident that enrichment by comminution alone cannot be used to increase the grade and that an alternative beneficiation method is required to achieve further upgrading.

### 2.2 Method

#### 2.2.1 Sample Characterization

In characterization studies, representative samples were collected from the ore and examined under a Nikon Eclipse 6400POL microscope (Figure 1a,b). Additionally, mineralogical examinations were performed using a Rigaku Miniflex 2 X-ray diffraction (XRD) device (Cu Kα radiation, 30 kV, 15 mA).

**Figure 1:**
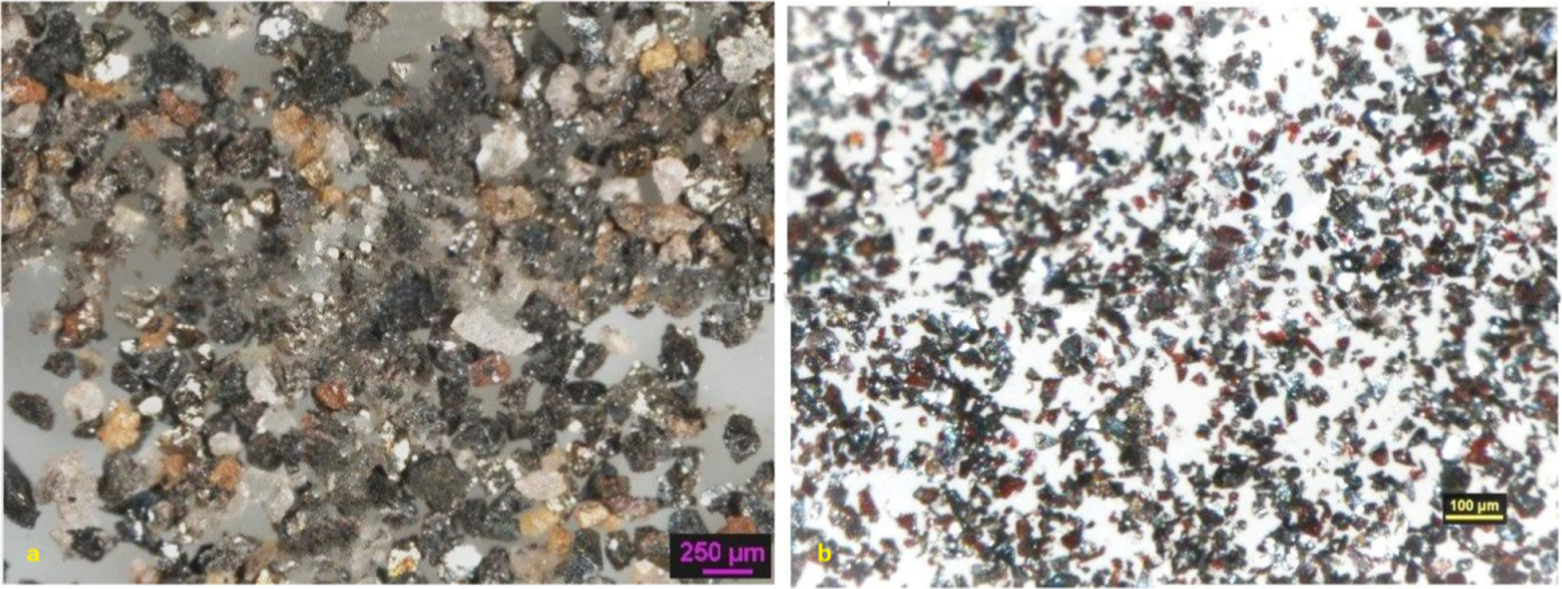
Optical microscope images: (a) 212–106 μm and (b) −106 μm.

Optical microscopy was employed to assess the liberation characteristics of micaceous iron oxide particles. For this analysis, the sample was separated into distinct size fractions. As illustrated in Figure 1, a notably higher concentration of these plate-like specularite particles was observed in the −106 μm size fraction.

The XRD patterns of the specularite ore are illustrated in Figure 2. The dominant phases were identified as hematite (Fe_2_O_3_) and quartz (SiO_2_). Characteristic diffraction peaks of hexagonal specularite were detected at approximately 24.1°, 33.1°, 35.6°, 49.4°, and 71.9° (2θ), corresponding to the (012), (104), (110), (024), and (119) crystallographic planes, respectively. The mineral α-Fe_2_O_3_ (hematite) exhibits a hexagonal crystal structure and belongs to the *R*3̅*c* space group.^53^ Consistent with these findings, previous researchers reported hematite reflections at comparable angles and planes.^53–55^ The intense, narrow, and sharply defined peaks further indicate that the sample is highly crystalline and structurally homogeneous.

**Figure 2:**
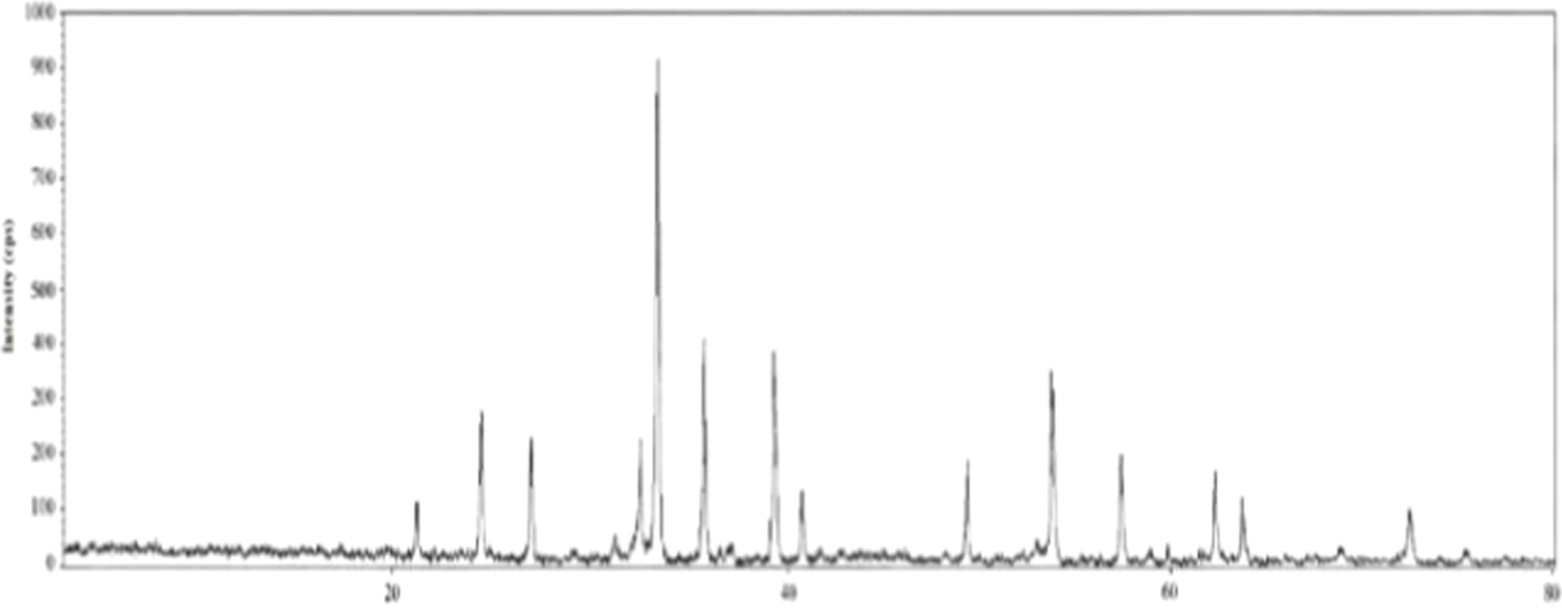
XRD diffractograms of specularite ore.

#### 2.2.2 Experimental Setup

A laboratory/pilot size Mozley MGS C900 with a nominal capacity of 150 kg/h, which consisted of an open-ended drum 500 mm in diameter and 600 mm in length, was used for the tests (Figure 3). In this study, certain parameters affecting the separation efficiency of the MGS, such as shaking frequency (4.8 cycles/s), shaking amplitude (10 mm), and feed flow rate (2 L/min), were kept constant. Other parameters, like drum speed, drum slope, and the amount of wash water, were varied according to experimental designs. All drum speed values reported in this study correspond to the actual rpm measured using a tachometer. The MGS unit is operated via a variable-speed controller, and an on-unit calibration chart is used to relate the controller set points to the drum rotational speed. The MGS tests were conducted as continuous tests, with at least 5 kg of feed in slurry form (25% solid ratio) introduced into the drum using a peristaltic pump. The system was allowed to run for a few minutes to reach a steady state. After 3 min of feeding, a small amount of samples was taken from the concentrate and tailings at 10 s intervals until the end of the test. These samples were combined to form the final sample for analysis, which was then assessed for Fe content. The weight and specularite recoveries were calculated based on the grades of feed, concentrate, and tailings.

**Figure 3:**
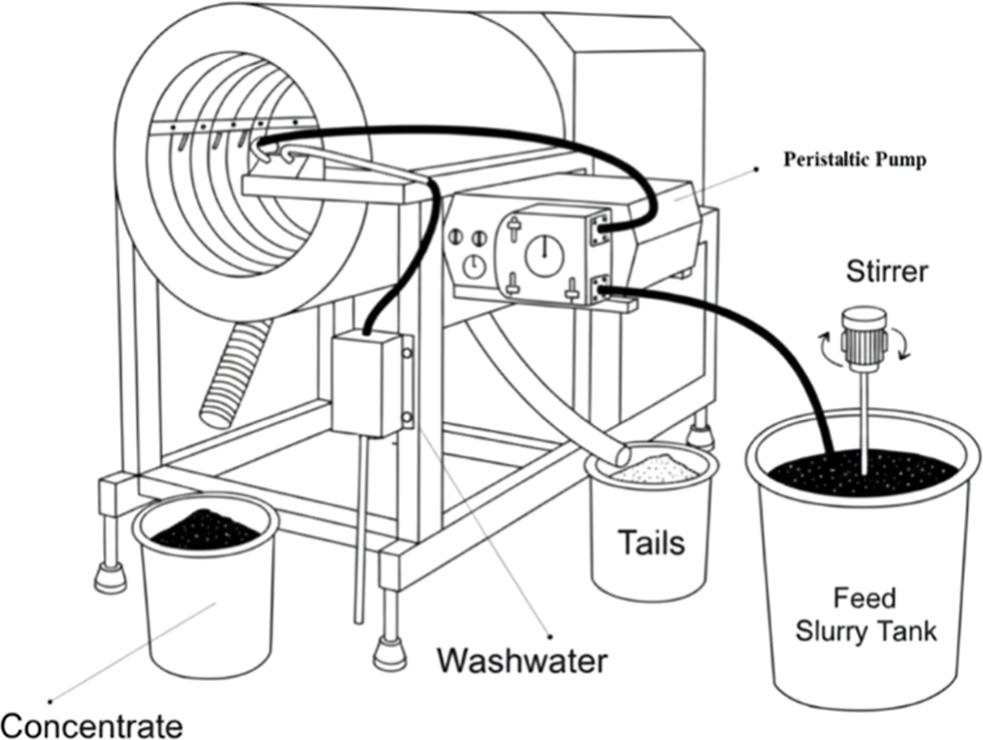
Multi-gravity separator experimental setup.

#### 2.2.3 Chemical Analysis

Total iron content was determined in accordance with ASTM E246, Test Method B—Stannous Chloride Reduction Dichromate Titration Method.^56^ Samples were dissolved through acid digestion using HCl, and the insoluble residue was filtered, ignited, treated for iron recovery, and combined with the main solution. Fusion decomposition was applied using a Na_2_CO_3_–Na_2_O_2_ flux, followed by water leaching. After the reduction process, the cooled solution was titrated with standard K_2_Cr_2_O_7_ using sodium diphenylamine sulfonate as the indicator. The end point was observed by a color change from green to bluish-green, followed by a final violet coloration.

#### 2.2.4 Design of Experiments and Response Surface Method

Response Surface Methodology (RSM) with central composite (CCD) and Box–Behnken (BBD) designs was used to empirically examine the relationship between process variables and the separation efficiency. Literature reports various studies examining how material characteristics and operating conditions influence the performance of the MGS. While some factors exert a strong impact, others show only marginal effects. Accordingly, the selection of parameters and their levels focused on those variables identified in previous research as having the greatest influence on grade and overall efficiency.^38,39,48,49,57^

Although MGS performance can be affected by multiple variables (e.g., shake frequency, shake amplitude, feed solids, feed flow rate, and tilt angle), optimization studies commonly focus on a small subset of the most influential controllable parameters while holding secondary factors constant to keep the design economical and interpretable. For example, Box–Behnken/RSM optimization of MGS performance has been reported using three major parameters such as drum speed, tilt angle, and shaking amplitude; similarly, regression and ANN models have been developed by selecting practical operating variables for prediction.^58–62^

The list of the independent variables (A, B, and C) with their coded and actual levels for CCD and BBD is presented in Table 2.

**Table 2:** Variables and Levels for BBD and CCD Experimental Designs

	symbol	BBD			CCD				
parameters		variables and range			variables and range				
		–1	0	1	-α	–1	0	1	+α
drum speed (rpm)	A	100	137.5	175	74.4	100	137.5	175	200.6
amount of wash water (L/min)	B	4	5.5	7	3	4	5.5	7	8
drum slope (°)	C	2	3.5	5	1	2	3.5	5	6

CCD provides robust estimates and handles missing data effectively, covering more factor-level combinations than BBD, which, while reliable at center points, performs poorly at corners due to the absence of design points.^63^ Moreover, the total number of experiments in BBD is considerably lower than in CCD, with 15 runs for BBD compared to 20 runs in the CCD method.

Response Surface Methodology (RSM) encompasses a set of statistical and mathematical techniques that are useful for developing, improving, and optimizing processes. The aim of a carefully designed experiment is to enhance a response influenced by multiple independent variables, with the output determined through a structured regression analysis based on the controlled values of these variables.^64,65^

Design of Experiments (DoE) and Response Surface Methodology (RSM) have been widely applied for optimizing gravity separation. DoE is valuable for obtaining maximum information from a limited number of systematically arranged experiments by simultaneously varying all process factors, while RSM provides a set of mathematical and statistical tools for constructing an experimental model that relates the response to the key process variables.^66^ Therefore, in this study, the DoE and RSM were jointly employed to effectively model and optimize the key process parameters.

A second-order polynomial equation was selected to fit the experimental results. This model represents the effects of process variables (A–C) and their interactions on the response variables (Fe grade and Fe recovery). The general form of the chosen model is represented as (eq 1) follows

1 $$yn=\beta_{0}+\beta_{1}x_{1}+\beta_{2}x_{2}+\beta_{3}x_{3}+\beta_{4}x_{1}^{2}+\beta_{5}x_{2}^{2}+\beta_{6}x_{3}^{2}+\beta_{7}x_{1}x_{2}+\beta_{8}X_{1}x_{3}+\beta_{9}x_{2}x_{3}$$

where yn is the predicted response; β_0_ is the model constant; β_1_, β_2_ and β_3_ are linear coefficients; β_4_, β_5_, and β_6_ are the quadratic coefficients; and β_7_, β_8_, and β_9_ are cross-product coefficients. Mathematical model equations were derived using experimental data and the mathematical software package Minitab 17 (Minitab Incorporation, State College, PA).

ANOVA was performed to assess the statistical significance, model adequacy, and parameter effects. Model verification included evaluation of *R* ^2^, adjusted *R* ^2^, predicted *R* ^2^, lack-of-fit tests, and residual diagnostics. Prediction accuracy was further assessed by using the Relative Standard Error (RSE).

A multiresponse desirability function approach was applied to simultaneously maximize Fe recovery and Fe grade. Both responses were treated as “larger-the-better” and assigned equal importance. Individual desirability functions were combined into an overall desirability index, which was then maximized to obtain the optimal settings of A, B, and C. Optimum conditions were determined separately for the BBD and CCD-based models. Finally, confirmation experiments were conducted at the respective optimum settings, and the experimentally obtained responses were compared to those predicted by the RSM models.

## 3 Results and Discussion

### 3.1 The Statistical Evaluation of the Model

The aim of this study was to evaluate the suitability of various models for optimizing the beneficiation process of specularite ore using MGS. Hence, BBD and CCD designs were tested. The design matrices are presented in Tables 3 and 4, respectively.

**Table 3:** Box–Behnken Experimental Design Matrix Using Three Levels and Three Factors and Their Predicted Results

experiment no.	A	B	C	actual values		predicted values	
				Fe (%)	recovery (%)	Fe (%)	recovery (%)
1	100	4	3.5	63.66	67.83	63.63	68.50
2	100	5.5	2	64.80	62.98	64.94	62.03
3	100	5.5	5	65.44	62.25	65.61	61.53
4	100	7	3.5	66.34	54.39	66.06	55.38
5	137.5	7	2	62.01	72.04	62.15	71.99
6	137.5	5.5	3.5	63.16	73.30	62.74	74.82
7	137.5	7	5	64.37	67.92	64.48	67.65
8	137.5	4	2	60.58	79.56	60.47	79.83
9	137.5	5.5	3.5	61.91	76.26	62.74	74.82
10	137.5	5.5	3.5	63.15	74.89	62.74	74.82
11	137.5	4	5	62.11	75.86	61.97	75.90
12	175	5.5	5	62.60	73.04	62.46	73.98
13	175	4	3.5	59.29	80.50	59.57	79.51
14	175	5.5	2	59.48	81.03	59.30	81.75
15	175	7	3.5	61.29	77.21	61.33	76.54

**Table 4:** Central Composite Experimental Design Matrix Using Three Levels and Three Factors and Their Predicted Results

experiment no.	A	B	C	actual values		predicted values	
				Fe (%)	recovery (%)	Fe (%)	recovery (%)
1	74.43	5.5	3.5	66.22	52.22	66.07	51.77
2	100	7	2	64.9	54.73	65.06	55.9
3	100	4	2	63.15	71.46	62.82	70.99
4	100	7	5	66.41	54.49	66.6	54.07
5	100	4	5	63.77	65.68	63.94	66.42
6	137.5	5.5	0.98	61.44	77.86	61.82	77.39
7	137.5	5.5	3.5	63.24	75.54	63.15	74.75
8	137.5	5.5	3.5	63.4	74.63	63.15	74.75
9	137.5	5.5	3.5	62.78	75.27	63.15	74.75
10	137.5	2.98	3.5	60.09	79.64	60.46	79.69
11	137.5	5.5	3.5	62.81	74.68	63.15	74.75
12	137.5	5.5	3.5	63.63	73.22	63.15	74.75
13	137.5	5.5	3.5	63.07	75.09	63.15	74.75
14	137.5	8.02	3.5	64.79	64.82	64.56	64.29
15	137.5	5.5	6	64.59	68.39	64.34	68.38
16	175	7	2	61.82	78.51	61.56	78.11
17	175	4	2	59.62	83.33	59.34	84.09
18	175	4	5	61.07	75.94	60.81	75.11
19	175	7	5	63.22	71.05	63.45	71.86
20	200.57	5.5	3.5	60.2	77.78	60.49	77.75

Tables 3 and 4 also show the predicted responses of the process to the variables considered. The actual model equation for grade and recovery of the specularite concentrates according to BBD is given in eqs 2 and 3, respectively.

2 Grade(%)=68.17−0.1508A+3.00B−1.33C

3 $$Recovery\left(\%\right)=14.5+0.945A-6.72B+5.08C-0.003148A^{*}A+0.0451A^{*}B$$

The actual model equation for grade and recovery of the specularite concentrates according to CCD is given in eqs 4 and 5, respectively.

4 Grade(%)=62.02−0.0581A+1.773B+0.097C

5 $$Recovery\left(\%\right)=32.25+0.7427A-4.90B+1.32C-0.002512A^{*}A-0.435B^{*}B-0.299C^{*}C+0.04047A^{*}B-0.01962A^{*}C$$

The graphs in Figures 4 and 5 show the actual values obtained from the experiments plotted against the predicted values for BBD and CCD, respectively. The models fit the data well, with the differences between the observed values and the model’s predicted values being relatively small and unbiased for both experimental designs. The predicted values are consistent with the experimental results. The coefficients of determination (*R* ^2^) for BBD obtained for the grade and recovery are 0.9771 and 0.9872, respectively. Also, for CCD, grade and recovery *R* ^2^ values were found as 0.9774 and 0.9942, respectively, indicating that the regression is significant for both designs.

**Figure 4:**
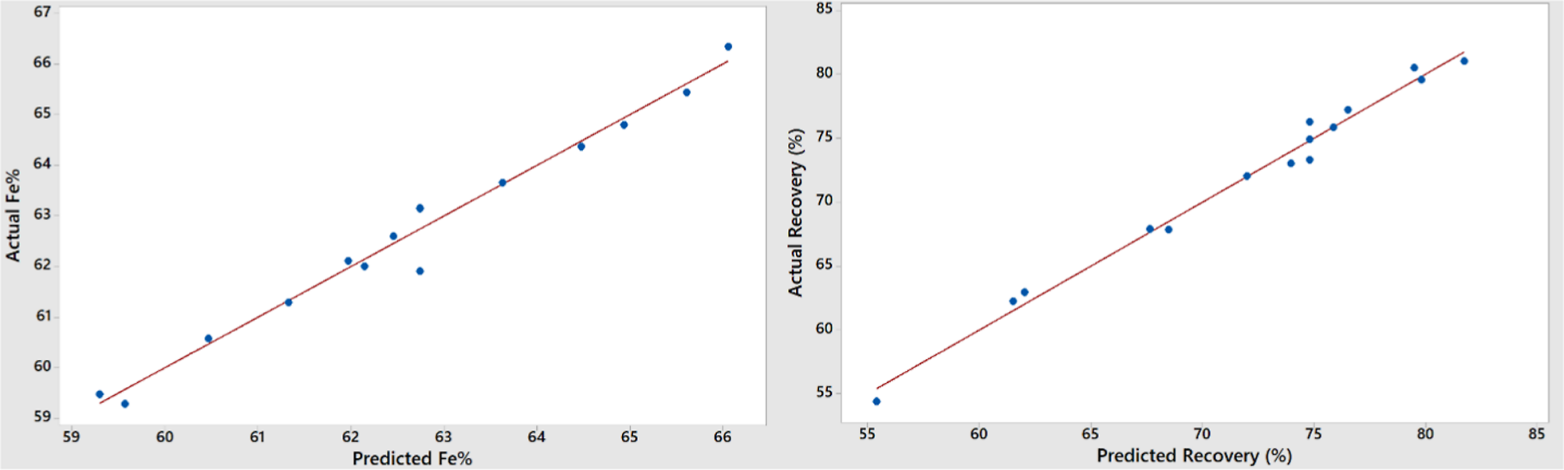
Relationship between actual and predicted values of the process responses, Fe grade, and recovery for BBD.

**Figure 5:**
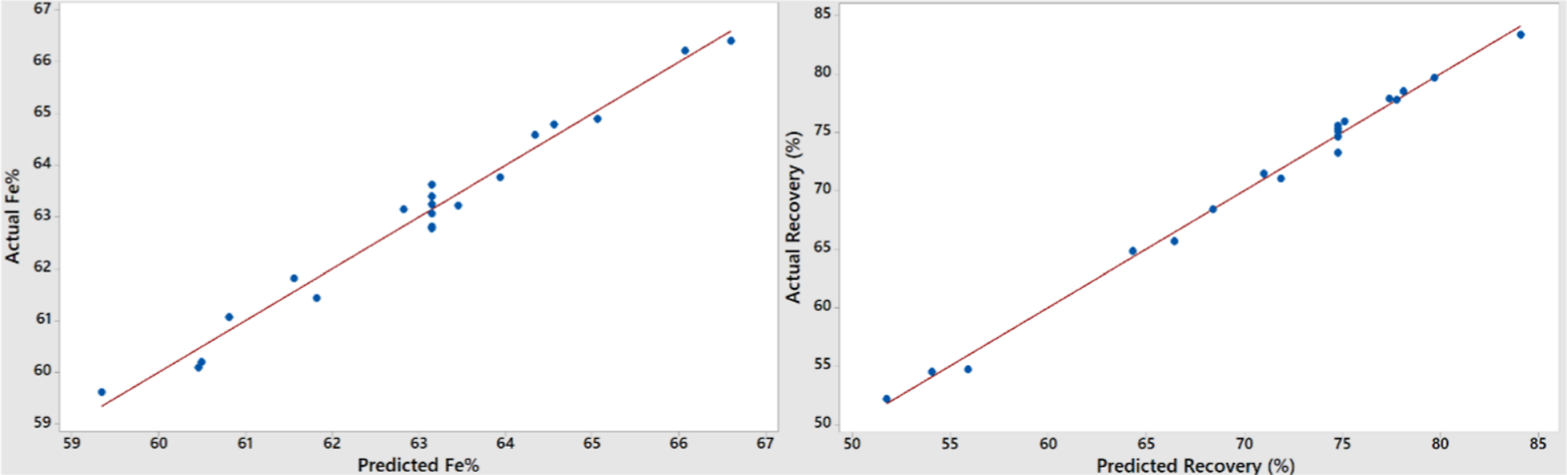
Relationship between actual and predicted values of the process responses, Fe grade, and recovery for CCD.

The significance test of model fit for the grade and recovery of the concentrates was conducted using Minitab 17 software based on analysis of variance (ANOVA). The results indicated that the models are significant, as the *F*-value is high and the Prob > *F* value is less than 0.05 (Table 5). The lack of fit values was higher than 0.05 for the grade and recovery model, suggesting that the lack of fit is not significant relative to the pure error for both models. The predicted *R* ^2^ values for the grade and recovery models are in reasonable agreement with the adjusted *R* ^2^ values, with the differences between these values being less than 0.2 for both models. The relative errors between the grade and recovery actual and predicted values were calculated as 0.52% and 1.23% for BBD and 0.46% and 0.99% for CCD, respectively, with all of the relative errors below the 5% threshold, further demonstrating the feasibility, reliability, and consistency of the developed models.^67–69^

**Table 5:** Analysis of Variance (ANOVA) Table Derived for the Grade and Recovery Models

	BBD		CCD	
	grade	recovery	grade	recovery
*R* ^2^	0.9771	0.9872	0.9774	0.9942
adjusted *R* ^2^	0.9358	0.9643	0.9570	0.9899
predicted *R* ^2^	0.8740	0.8711	0.8767	0.9655
*F*-value	23.69	42.98	48.04	189.20
*p*-value	0.001	0.001<	0.001<	0.001<
lack of fit	0.885	0.568	0.282	0.327

The estimated coefficient values for the main effects of the process variables, calculated using eqs 2–5, are presented in Tables 6 and 7. These values indicate that drum speed (A) negatively affects the Fe grade of the concentrate, while the amount of wash water (B) and drum slope (C) have positive effects. Conversely, for recovery, drum speed (A) has a positive effect, whereas the amount of wash water (B) and drum slope (C) have negative effects within the tested range of parameter levels for both designs.

**Table 6:** Estimated Coefficient Values for the Parameter and Parameter Interaction Effects for BBD

	grade (%)			recovery (%)		
	coefficient estimate	*F*-value	*p*-value	coefficient estimate	*F*-value	*p*-value
A	–2.197	142.3	0	8.042	252.49	0
B	1.047	32.31	0.002	–4.023	63.2	0.001
C	0.956	26.94	0.003	–2.068	16.7	0.009
A*A	0.359	1.75	0.243	–4.428	35.33	0.002
B*B	–0.452	2.78	0.156	–0.409	0.3	0.607
C*C	–0.019	0	0.948	–0.566	0.58	0.482
A*B	–0.169	0.42	0.545	2.538	12.57	0.016
A*C	0.621	5.68	0.063	–1.818	6.45	0.052
B*C	0.207	0.63	0.464	–0.106	0.02	0.888

**Table 7:** Estimated Coefficient Values for the Parameter and Parameter Interaction Effects for CCD

	grade (%)			recovery (%)		
	coefficient estimate	*F*-value	*p*-value	coefficient estimate	*F*-value	*p*-value
A	–1.657	243.84	0	7.722	961.78	0
B	1.219	131.96	0	–4.583	338.4	0
C	0.754	50.26	0	–2704	117.39	0
A*A	0.045	0.19	0.67	–3.532	212.47	0
B*B	–0.227	4.83	0.053	–0.979	16.26	0.002
C*C	–0.023	0.05	0.831	–0.673	7.59	0.02
A*B	–0.005	0	0.972	2.276	48.95	0
A*C	0.09	0.42	0.531	–1104	11.51	0.007
B*C	0.105	0.57	0.466	0.684	4.42	0.062

Tables 6 and 7 show that the interactions between variables, for BBD, the interaction between drum speed (A) and drum slope (C) for grade, and also the interaction between drum speed (A) and amount of wash water (B) for recovery data, are significant, while other interactions are not significant for the model. When examining the interactions between parameters according to CCD, while grade values do not significantly affect the model, interactions between drum speed with wash water (A*B) and drum speed with slope (A*C) are significant for recovery. On the other hand, drum speed (A) has the highest quadratic effect on the concentrate Fe grade for both designs.

### 3.2 The Effect of Process Variables on Grade and Recovery

The effects of drum speed (A), wash water flow rate (B), and drum slope (C) on the predicted Fe grade and Fe recovery are illustrated by the three-dimensional response surfaces in Figures 6–11 for BBD (Figures 6–8) and CCD (Figures 9–11). These surfaces provide a visual representation of the trade-offs between grade and recovery within the studied operating ranges.

**Figure 6:**
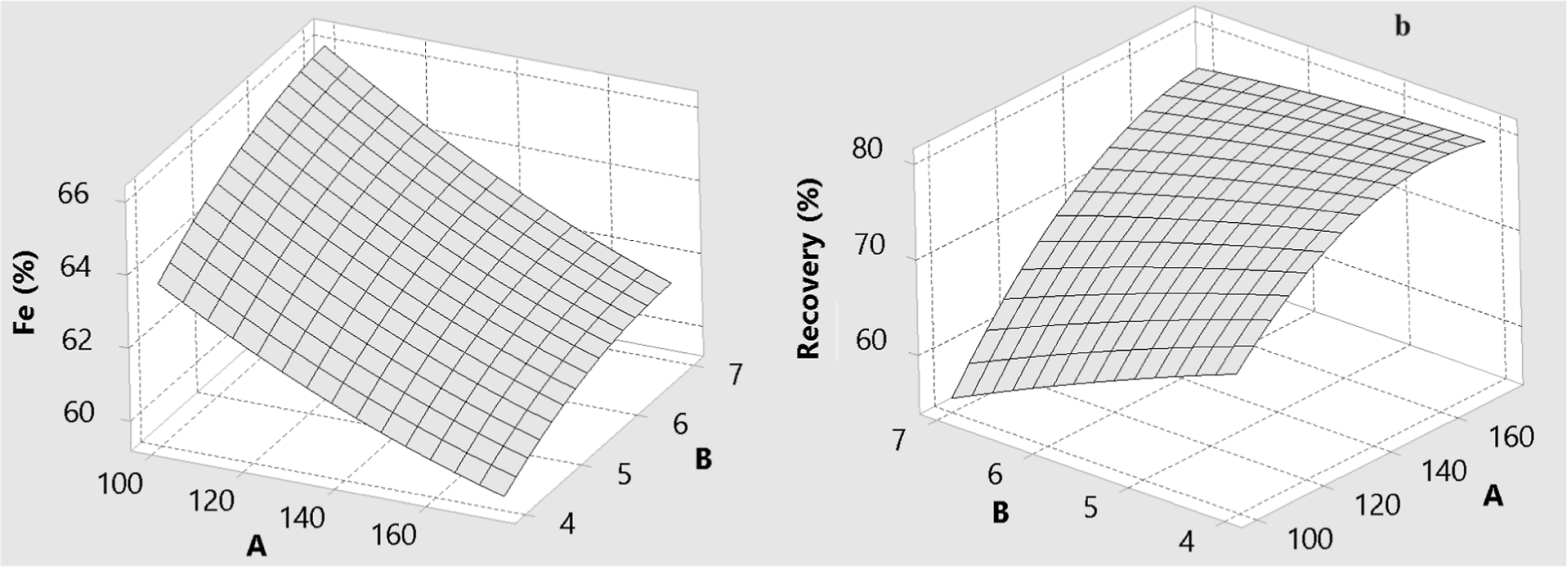
Effect of drum speed (A) and amount of wash water (B) for MGS concentrate for BBD: (a) Fe grade and (b) recovery.

**Figure 7:**
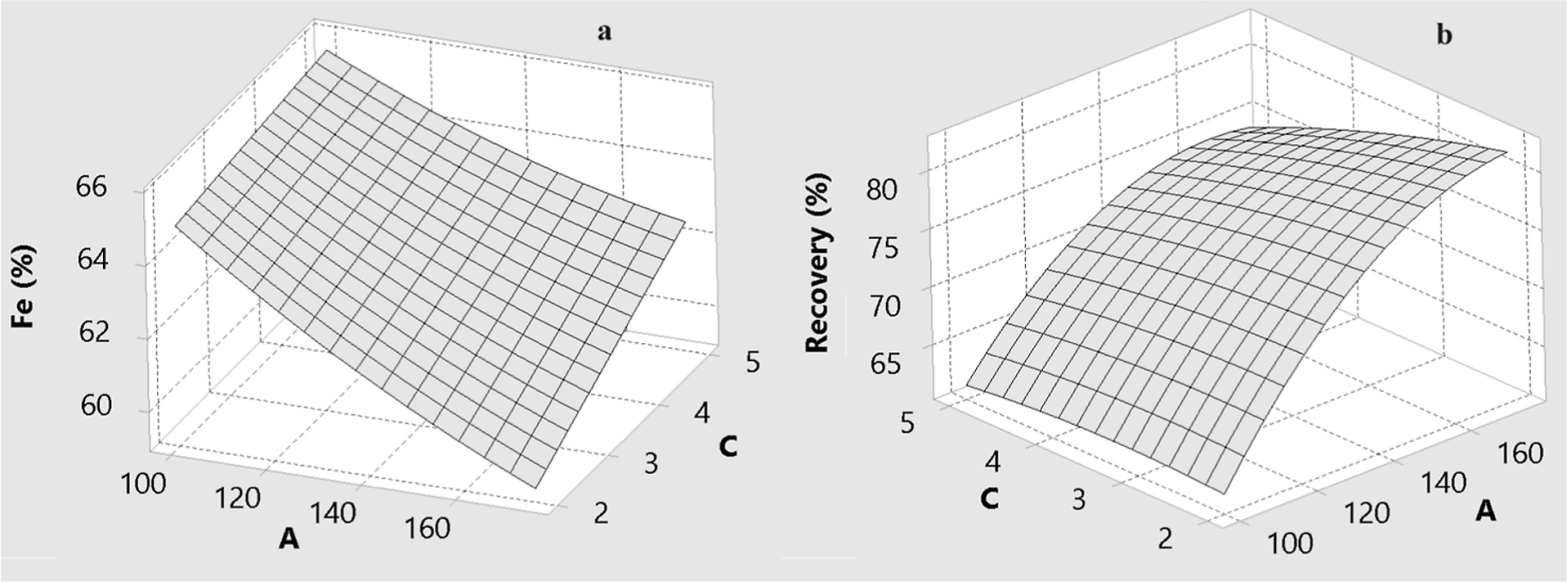
Effect of drum speed (A) and drum slope (C) for MGS concentrate for BBD: (a) Fe grade and (b) recovery.

**Figure 8:**
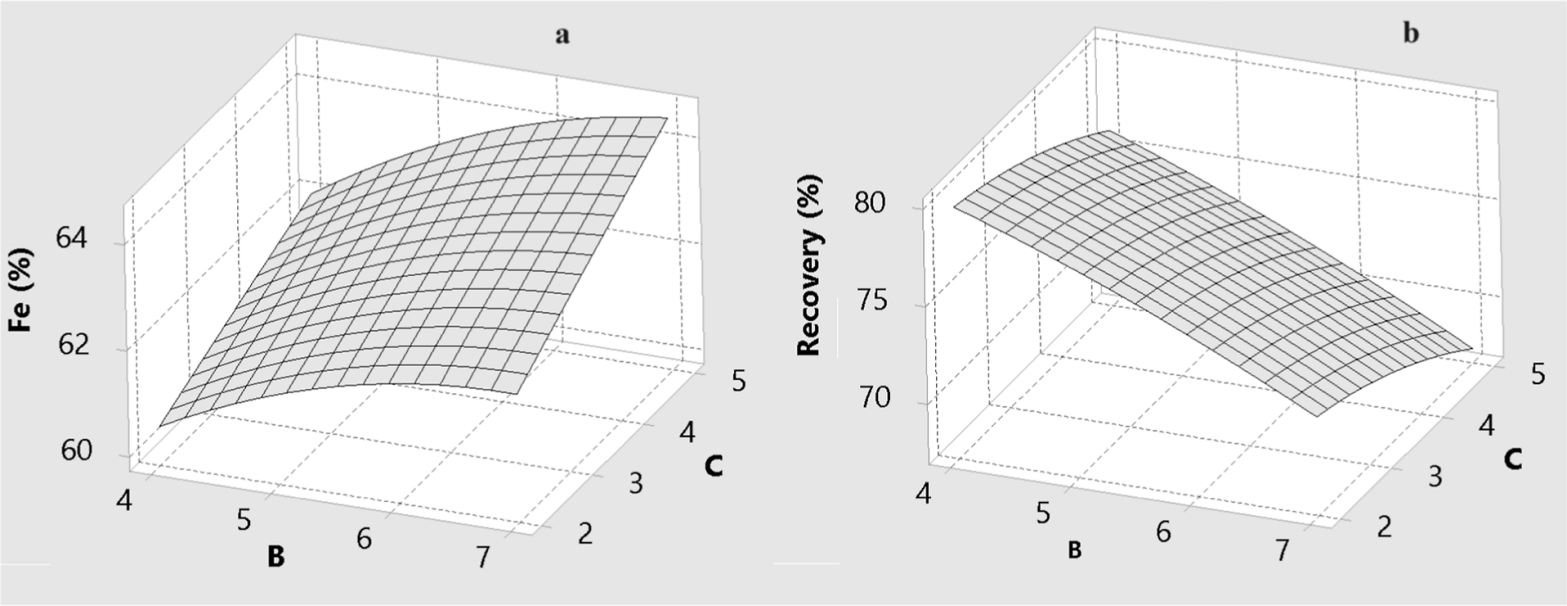
Effect of the amount of wash water (B) and drum slope (C) for MGS concentrate for BBD: (a) Fe grade and (b) recovery.

**Figure 9:**
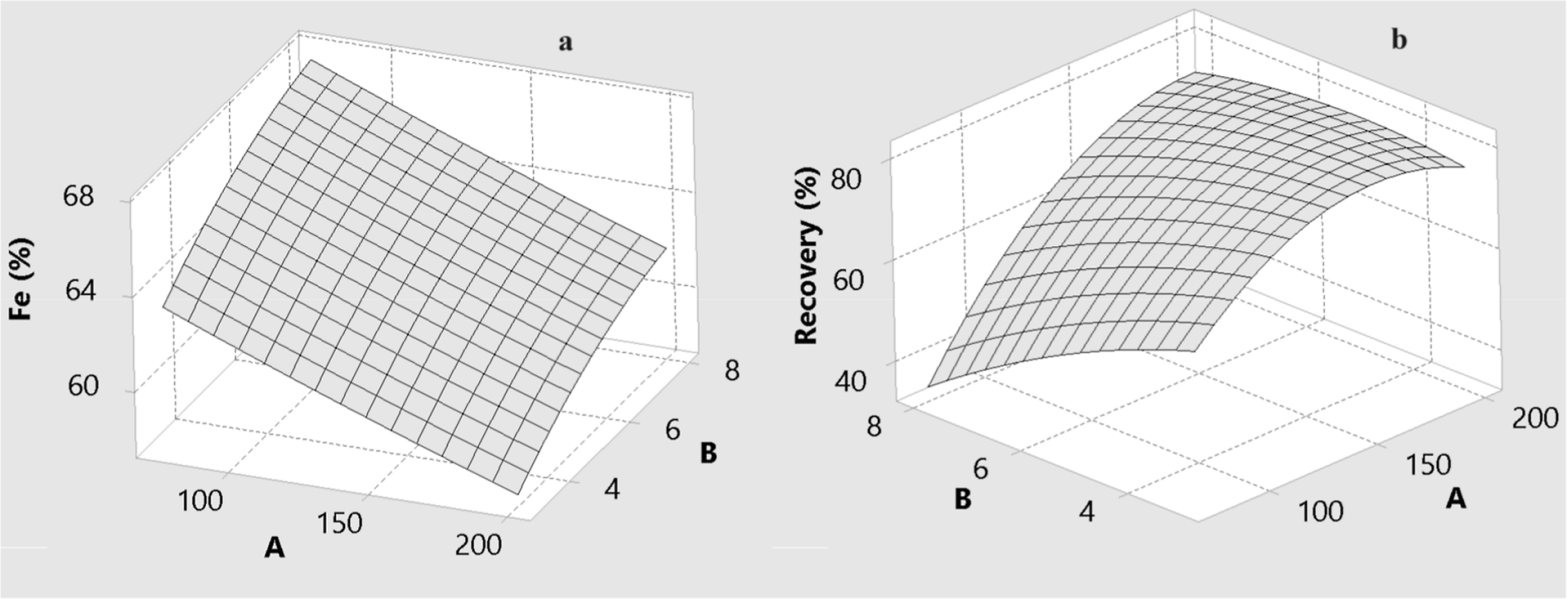
Effect of drum speed (A) and amount of wash water (B) for MGS concentrate for CCD: (a) Fe grade and (b) recovery.

**Figure 10:**
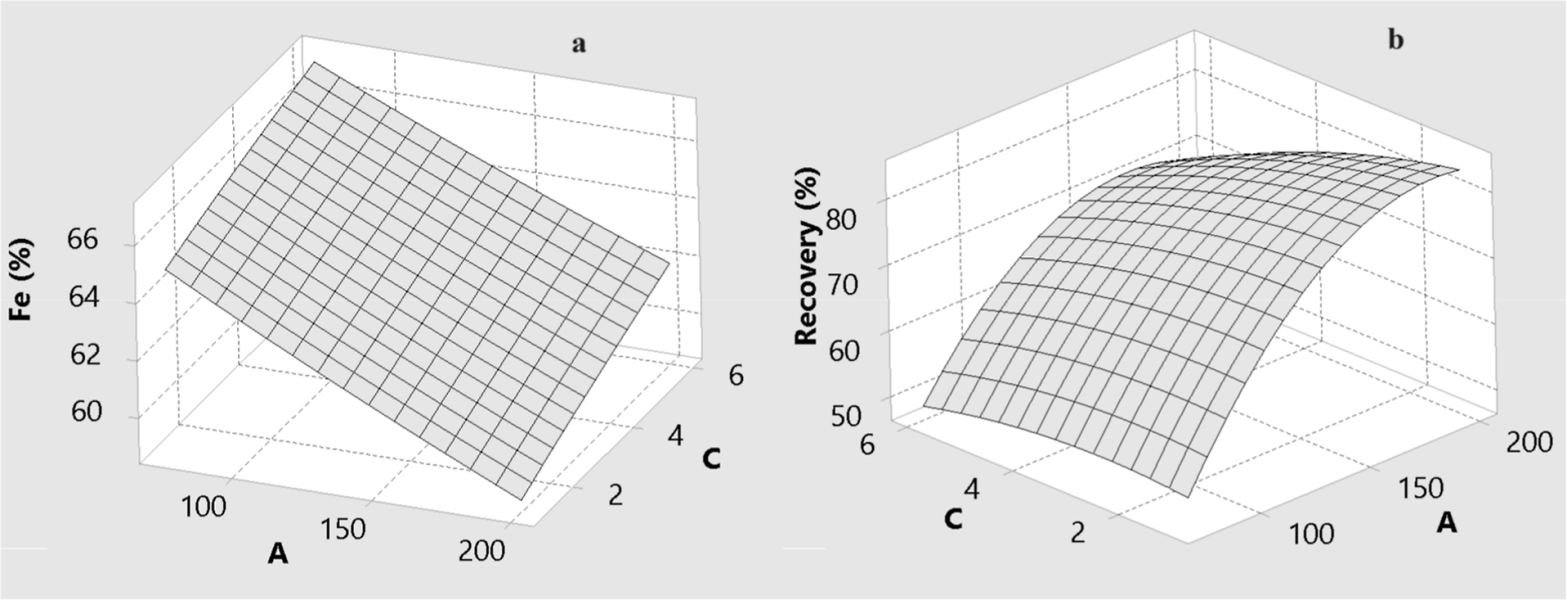
Effect of drum speed (A) and drum slope (C) for MGS concentrate for CCD: (a) Fe grade and (b) recovery.

**Figure 11:**
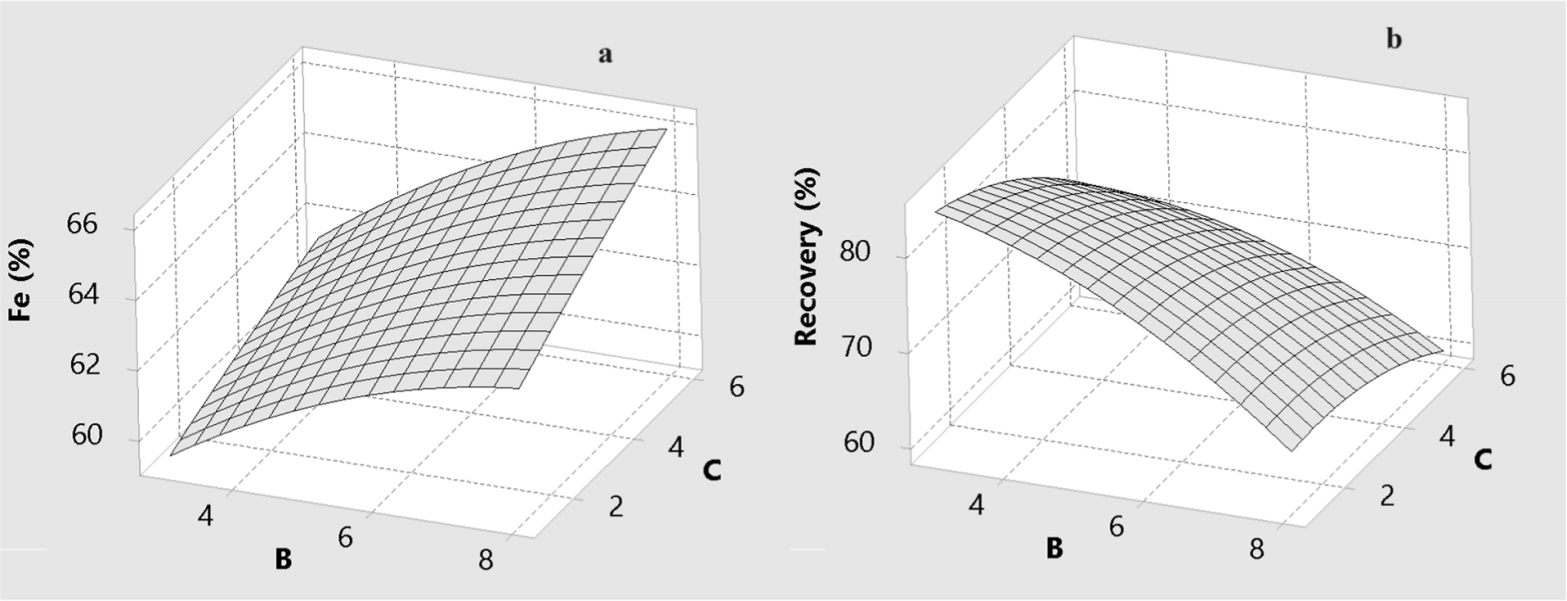
Effect of amount of wash water and drum slope for MGS concentrate for CCD: (a) Fe grade and (b) recovery.

Figures 6 and 7 show the combined effects of drum speed with wash water (A and B) and drum speed with drum slope (A and C) for the BBD. In both designs, Fe recovery increases with increasing drum speed, whereas Fe grade tends to decrease at higher speeds. Drum speed is therefore identified as the most influential factor for Fe recovery. As rotational speed increases, the centrifugal force acting on dense specularite particles becomes much greater than the gravitational force, which enhances density-based stratification and promotes the migration of heavy particles toward the compact solids bed in the collection zone.

However, at excessively high speeds, the separation selectivity deteriorates. Stronger shear and turbulence within the thin film disturb the settling of partially liberated or fine specularite particles. At the same time, lamellar gangue minerals such as muscovite and fine quartz can become entrained in the concentrate due to their plate-like morphology and the increased fluid drag acting on their surfaces. Consequently, the Fe grade decreases beyond the optimum speed range, even though recovery continues to rise. These observations highlight a nonlinear response pattern: moderate increases in drum speed improve recovery, but further increases lead to diminishing returns in recovery and a measurable loss in grade due to entrainment and turbulence, in agreement with previous work on enhanced gravity separation.^70^

As can be seen from Figures 8 and 9, an increase in the level of wash water significantly decreases the recovery of specularite. Wash water regulates the hydraulic wash of the film surface, influencing both grade and recovery. Low to moderate wash water flow rates encourage cleaner separation because the thin water layer allows lighter particles to be effectively displaced into the tailing stream. This improves the Fe grade by minimizing gangue contamination.

Nevertheless, beyond the optimal threshold, increased hydraulic velocity begins to displace fine or weakly liberated specularite particles, causing Fe losses in the tailings. This selective flushing effect is more pronounced for plate-like minerals with higher surface area-to-mass ratios. Consequently, while wash water is essential for improving grade, excessive amounts reduce recovery. Similar results were also observed in a previous study.^71^ The strong dependence of grade on wash water and its interaction with drum speed confirms this parameter’s critical role in controlling film stability and fine particle transport.

Figures 10 and 11 indicate that the drum slope primarily governs the axial transport and slurry residence time within the MGS. An increasing slope promotes rapid movement of material along the drum surface, which decreases the time available for stratification based on density contrasts. This naturally reduces Fe recovery, particularly for particles near the cut-size threshold.

Conversely, moderate slopes assist in the removal of low-density gangue minerals, thereby improving the grade. This secondary improvement reflects the enhanced removal of silicate particles that are less likely to settle under the influence of centrifugal forces. Although the drum slope exerts comparatively weaker main effects than drum speed and wash water, its influence becomes more pronounced when considered alongside interactive effects. This substantiates previous findings in the literature.^72,73^

### 3.3 Analysis of Interactive Effects

Enhanced gravity separators such as the MGS are strongly affected by interaction effects because centrifugal and hydraulic forces act simultaneously on particles of differing size, density, and liberation. The ANOVA results in Tables 6 and 7, together with the response surfaces in Figures 6–11, highlight several key two-factor interactions.

#### 3.3.1 Interaction between Drum Speed and Wash Water

The interaction between drum speed and wash water (A,B) is among the most important, particularly for Fe recovery. At low wash water levels, an increase in drum speed leads to a substantial improvement in recovery, as centrifugal forces dominate and dense particles are efficiently driven toward the concentrate bed. Under these conditions, hydraulic drag is relatively weak, so few valuable particles are flushed into the tailings.

When the wash water flow rate is high, however, the beneficial effect of increasing drum speed is strongly moderated. Elevated hydraulic drag counteracts the settling tendency of dense specularite particles, and the intensified turbulence enhances entrainment of partially liberated or fine particles into the tailings stream. In practical terms, a high drum speed cannot fully compensate for excessive wash water. This interaction produces a curved response surface with a relatively narrow window of operating conditions in which grade and recovery are balanced; outside this window, either entrainment of the gangue (low wash water) or flushing of valuable particles (high wash water) becomes dominant.

#### 3.3.2 Interaction between Drum Speed and Drum Slope

The A–C interaction is particularly relevant for Fe recovery. The combined increase in drum speed and drum slope resulted in the steepest decline in recovery. Mechanistically, high drum speed increases shear and turbulence in the film, whereas an increased slope accelerates axial transport of the slurry. When both parameters are high, particle residence time is significantly reduced and the system departs from settling equilibrium, so that even relatively dense particles are prematurely conveyed to the tailings.

By contrast, moderate drum speeds combined with low or moderate slopes provide sufficient centrifugal force for stratification without causing excessive axial transport. In this region, dense specularite particles have enough time to migrate into the solids bed, while most of the low-density gangue particles are washed away. This combination forms part of the optimal operating window identified in the response-surface plots and in the subsequent desirability-based optimization.

#### 3.3.3 Interaction between the Wash Water Flow Rate and Drum Slope

The interaction between the wash water flow rate and drum slope (B–C) mainly affects Fe recovery and exhibits a weaker but still meaningful effect compared with the A–B and A–C interactions. Simultaneous increases in B and C tend to produce pronounced negative effects on recovery. Both parameters promote the transport of particles toward the tailings: wash water through hydraulic drag and the drum slope through enhanced axial conveying. When combined, these two mechanisms accelerate the discharge of fine and weakly liberated specularite, leading to measurable recovery losses.

Nevertheless, the response surfaces also indicate parameter regions where the B–C interaction has a beneficial effect on Fe grade. At moderate slopes and moderate wash water rates, gangue removal is maximized without excessive flushing of valuable minerals. In this operating window, the interaction between hydraulic washing and axial transport enhances the rejection of low-density silicate particles and improves the concentrate quality. Overall, the interaction analysis confirms that drum speed exerts the strongest influence on recovery, while wash water and drum slope play crucial supporting roles that must be carefully balanced to control entrainment, flushing, and residence time.

### 3.4 Response Optimization

Process optimization was conducted using multiresponse desirability functions to simultaneously maximize Fe grade and Fe recovery to provide a more realistic, industry-relevant optimum regarding optimization methodology. Unlike single-response maximization, multiresponse desirability provides a more holistic and industry-relevant determination of optimum operating conditions.

Both the BBD and CCD models were optimized within their respective design spaces.

The optimization was based on quadratic regression models for Fe grade and Fe recovery and on a composite desirability index (D) integrating both objectives. Individual desirability functions d_1_ and d_2_ were defined for Fe grade and Fe recovery, respectively, using a “larger-the-better” formulation. The overall desirability was calculated as the geometric mean of individual desirabilities (eq 6)­

6 Overalldesirability=(d1×d2)(d1×d2)^(1/2)

Both responses were assigned equal weights due to their industrial significance. The resulting desirability surfaces revealed a ridge-type region in which improvements in grade require a gradual sacrifice in recovery. The CCD design produced a narrower and more sharply defined high-desirability zone, consistent with its higher F-values and lower prediction uncertainty. The optimum response values and corresponding operating conditions are summarized in Table 8.

**Table 8:** Optimization Outputs

design	optimum grade (%)	optimum recovery (%)	drum speed (rpm)	wash water (L/min)	drum slope (°)	composite desirability (*D*)
BBD	63.79	71.83	140.15	5.79	5.00	0.842
CCD	64.00	71.47	143.23	6.24	4.53	0.891

CCD yielded a slightly higher composite desirability (*D* = 0.891) compared with that of BBD (*D* = 0.842), indicating superior overall optimization performance. Nevertheless, the optimum Fe grades and recoveries obtained from the two designs were very similar (Table 8), which confirms the robustness of the underlying relationships between the process variables and the responses.

The predictive quality of the optimum solutions was assessed by comparing the model predictions with the results of validation experiments conducted at the respective optimum settings (Table 9). For the BBD-based optimum, the confirmation run yielded a concentrate assaying 63.58% Fe with 72.38% recovery, while for the CCD-based optimum, 63.97% Fe and 72.05% recovery were obtained. All experimental values fall within the 95% confidence intervals of the predicted responses, demonstrating the accuracy of the optimized models.

**Table 9:** Optimization Test Results

	BBD		CCD			
parameter	value	CI (95%)	value	CI (95%)		
Fe (%)	63.79	62.94	64.64	64.00	63.63	64.36
recovery (%)	71.83	69.50	74.16	71.47	70.62	72.33
A	140.15			143.23		
B	5.79			6.24		
C	5.00			4.53		

Overall, the optimization study shows that the CCD design outperformed the BBD in terms of precision, composite desirability, and validation accuracy. Despite minor differences in the optimum parameter sets, both designs converged to nearly identical operating windows, indicating that the optimum region is well-defined. In practical terms, moderate drum speeds and wash water flow rates provide the best balance between hydraulic cleaning and centrifugal separation, whereas drum slope primarily controls slurry residence time and fine particle washout, exerting a stronger influence on recovery than on grade.

## 4 Conclusion

This study presents a comprehensive investigation into the optimization of multigravity separator (MGS) operating parameters for the beneficiation of specularite ore, integrating mineralogical characterization, statistical modeling, and multiresponse optimization. The analysis incorporates deeper scientific interpretation, expanded diagnostics, and a rigorous comparison with previous studies. Drum speed, wash water flow rate, and drum slope were identified as the primary factors governing separation dynamics. Drum speed exerted the strongest influence on recovery, whereas the wash water flow rate most significantly affected grade. Mineralogical analysis revealed platy, weakly liberated specularite grains in the −106 μm fraction, explaining the moderate variation in Fe grade and the sensitivity to wash water and residence time. The interplay between centrifugal forces and hydraulic flushing was found to be crucial in determining the separation selectivity.

Both Box–Behnken Design (BBD) and Central Composite Design (CCD) produced robust predictive models with *R* ^2^ values exceeding 0.97. CCD demonstrated superior statistical precision, higher *F*-values, and lower prediction errors, confirming its advantage for process optimization. Multiresponse desirability analysis indicated that comparable optimum grade and recovery values could be achieved using either experimental design, highlighting the stability of the process. The CCD, however, achieved a higher composite desirability, defining a more precisely constrained optimal region. Confirmation experiments closely matched model predictions with deviations of less than 1%, reinforcing the reliability of the models and the appropriateness of the optimized conditions.

The results of the numerical optimization in the range of the experimental data showed that it is possible to produce a concentrate assaying 63.97% Fe with 72.05% recovery. Although these recovery values are moderate relative to conventional iron ore beneficiation targets, they are fully acceptable for micaceous iron oxide (MIO) pigment production, where particle lamellarity must be preserved, overgrinding reduces pigment quality, and economic value is driven by product morphology rather than maximum metal recovery. The optimized operating conditions offer a balanced compromise among recovery, grade, and product characteristics critical for industrial applications. This study provides several novel contributions to the mineral processing literature. It presents the first comparative assessment of BBD and CCD for MGS optimization applied to specularite ore, integrates mineralogical evidence, statistical modeling, and experimental validation to explain separation behavior mechanistically, and establishes a predictive and validated optimization framework suitable for industrial decision-making.

Further studies are recommended to enhance understanding of specularite beneficiation, including preliminary screening of additional parameters using Plackett–Burman or Taguchi methods, exploration of alternative particle preparation techniques to improve liberation without altering lamellar morphology, evaluation of MGS performance at pilot or industrial scales under varying feed rheology, integration with magnetic separation or flotation to improve overall recovery, and advanced computational modeling (CFD-DEM) to visualize particle behavior under different hydrodynamic regimes. Overall, the results demonstrate that the MGS, when properly optimized, can produce specularite concentrates that meet industrial quality requirements, and the predictive modeling framework established herein provides a strong foundation for future process development.

## Data Availability

All of the material is owned by the authors and/or no permissions are required. The data underlying this study are available in the published article.

## Abbreviations

ANOVA: analysis of variance
ASTM: American Society for Testing and Materials
BBD: Box–Behnken Design
CCD: central composite design
CI: confidence interval
DoE: design of experiments
EGS: enhanced gravity separators
HGMS: high-gradient magnetic separator
MGS: multi-gravity separator
MIO: micaceous iron oxide
RSE: relative standard error
RSM: response surface methodology
